# Results of Genome-Wide Analyses on Neurodevelopmental Phenotypes at Four-Year Follow-Up following Cardiac Surgery in Infancy

**DOI:** 10.1371/journal.pone.0045936

**Published:** 2012-09-25

**Authors:** Daniel S. Kim, Ian B. Stanaway, Ramakrishnan Rajagopalan, Judy C. Bernbaum, Cynthia B. Solot, Nancy Burnham, Elaine H. Zackai, Robert R. Clancy, Susan C. Nicolson, Marsha Gerdes, Deborah A. Nickerson, Hakon Hakonarson, J. William Gaynor, Gail P. Jarvik

**Affiliations:** 1 Department of Medicine, Division of Medical Genetics, University of Washington School of Medicine, Seattle, Washington, United States of America; 2 Department of Genome Sciences, University of Washington School of Medicine, Seattle, Washington, United States of America; 3 Division of General Pediatrics, Children’s Hospital of Philadelphia, Philadelphia, Pennsylvania, United States of America; 4 Center for Childhood Communication, Children’s Hospital of Philadelphia, Philadelphia, Pennsylvania, United States of America; 5 Division of Cardiothoracic Surgery, Children’s Hospital of Philadelphia, Philadelphia, Pennsylvania, United States of America; 6 Division of Genetics, Children’s Hospital of Philadelphia, Philadelphia, Pennsylvania, United States of America; 7 Division of Neurology, Children’s Hospital of Philadelphia, Philadelphia, Pennsylvania, United States of America; 8 Division of Cardiothoracic Anesthesiology, Children’s Hospital of Philadelphia, Philadelphia, Pennsylvania, United States of America; 9 Division of Psychology, Children’s Hospital of Philadelphia, Philadelphia, Pennsylvania, United States of America; 10 Center for Applied Genomics, Children’s Hospital of Philadelphia, Philadelphia, Pennsylvania, United States of America; 11 Department of Pediatrics, University of Pennsylvania School of Medicine, Philadelphia, Pennsylvania, United States of America; Vanderbilt University, United States of America

## Abstract

**Background:**

Adverse neurodevelopmental sequelae are reported among children who undergo early cardiac surgery to repair congenital heart defects (CHD). *APOE* genotype has previously been determined to contribute to the prediction of these outcomes. Understanding further genetic causes for the development of poor neurobehavioral outcomes should enhance patient risk stratification and improve both prevention and treatment strategies.

**Methods:**

We performed a prospective observational study of children who underwent cardiac surgery before six months of age; this included a neurodevelopmental evaluation between their fourth and fifth birthdays. Attention and behavioral skills were assessed through parental report utilizing the Attention Deficit-Hyperactivity Disorder-IV scale preschool edition (ADHD-IV), and Child Behavior Checklist (CBCL/1.5-5), respectively. Of the seven investigated, three neurodevelopmental phenotypes met genomic quality control criteria. Linear regression was performed to determine the effect of genome-wide genetic variation on these three neurodevelopmental measures in 316 subjects.

**Results:**

This genome-wide association study identified single nucleotide polymorphisms (SNPs) associated with three neurobehavioral phenotypes in the postoperative children ADHD-IV Impulsivity/Hyperactivity, CBCL/1.5-5 PDPs, and CBCL/1.5-5 Total Problems. The most predictive SNPs for each phenotype were: a *LGALS8* intronic SNP, rs4659682, associated with ADHD-IV Impulsivity (*P* = 1.03×10^−6^); a *PCSK5* intronic SNP, rs2261722, associated with CBCL/1.5-5 PDPs (*P* = 1.11×10^−6^); and an intergenic SNP, rs11617488, 50 kb from *FGF9*, associated with CBCL/1.5-5 Total Problems (*P* = 3.47×10^−7^). 10 SNPs (3 for ADHD-IV Impulsivity, 5 for CBCL/1.5-5 PDPs, and 2 for CBCL/1.5-5 Total Problems) had p<10^−5^.

**Conclusions:**

No SNPs met genome-wide significance for our three neurobehavioral phenotypes; however, 10 SNPs reached a threshold for suggestive significance (p<10^−5^). Given the unique nature of this cohort, larger studies and/or replication are not possible. Studies to further investigate the mechanisms through which these newly identified genes may influence neurodevelopment dysfunction are warranted.

## Introduction

Congenital heart defects (CHDs) are the most common human birth defect, with an incidence of 8 per 1000 live births (30,000–40,000 cases annually in the United States). Approximately one-third of children with CHD require early surgical intervention. Improved surgical outcomes and intensive care have resulted in longer life expectancies for many subjects with CHD. However, studies have identified adverse neurological and functional outcomes in many survivors of neonatal and infant cardiac surgery [1], [2], [3], [4]. Of these, neurodevelopmental dysfunction is the most common outcome in survivors of surgical palliation of CHD [5]. Within our cohort, 30% and 22% of 381 subjects who underwent cardiac surgery in infancy scored in the clinically significant range for inattention and hyperactivity/impulsivity, respectively, for the Attention-Deficit/Hyperactivity Disorder-IV scale, preschool edition (ADHD-IV), [6] at five years of age [7]. In addition, 15% of the cohort was in the clinically significant range for pervasive developmental problems (PDPs) [7] as determined by the Child Behavior Checklist (CBCL) [8] at five years of age. Follow-up on this group of children between ages five and 10 years of age further identified 30% and 29% of 109 children who underwent cardiac surgery in infancy as having clinically significant scores for the Attention-Deficit Hyperactivity Disorder IV Scale (ADHD-IV) of inattention and hyperactivity/impulsivity, respectively, as ascertained by both reports from parents and/or teachers [9]. Moreover, 49% of the 109 subjects were receiving some sort remedial academic services and 15% had been assigned to a special education classroom.

Apolipoprotein E, coded by the *APOE* gene, is an important regulator of cholesterol metabolism and also a neuroresiliency gene. Prior work in this cohort of 550 neonates with CHD treated surgically in the first 6 months of life identified an association between the *APOE* ε2 allele and poorer neurodevelopmental outcomes of 244 children evaluated at 1 year of age survivors [10]. Of the original cohort, 381 underwent a detailed neurodevelopmental evaluation at 4 to 5 years of age. The *APOE* ε2 allele was associated with increased behavioral problems, impaired social interactions, and restricted behavior patterns at age 4–5 years [7], in addition to predicting poorer outcomes at 1 year of age [10].

With regard to genetics, ADHD is a well-studied neurodevelopmental outcome. A variable number tandem repeat (VNTR) of 7 alleles in the dopamine D4 receptor gene (*DRD4*) and a 148-bp microsatellite repeat in the dopamine D5 receptor gene (*DRD5*) have been consistently associated with ADHD [11], [12], [13], [14], [15], [16]. A VNTR polymorphism in the 3′-untranslated region (UTR) of *DAT1* (also known as *SLC6A3*), a carrier of dopamine that removes it from the synaptic cleft, has also been repeatedly associated with ADHD [11], [12], [15], [17], [18]. More recently, genome-wide association studies (GWAS) have identified SNPs in *CDH13, GFOD1,* and *TLL* that may be associated with ADHD, although replication was not present or not attempted in these studies [19], [20], [21].

A genome-wide association study of neurodevelopmental outcomes in a cohort of children who underwent major cardiac surgery has not been previously reported. Thus, our goal with this study was to conduct a GWAS to provide an unbiased assessment for common genetic variants that contribute strongly to common neurodevelopmental phenotypes in our unique cohort of children.

## Methods

### Ethics Statement

Subjects were collected at the Children’s Hospital of Philadelphia (CHOP) on a protocol approved by the Institutional Review Boards of CHOP and the University of Washington from 10/1998–04/2003. Informed, written consent was obtained from parents or guardians of all the subjects.

### Study Design

This was genome-wide analysis of a previously described prospective cohort [7], [10] to identify gene regions potentially affecting neurobehavioral outcomes for preschool-aged subjects (4–5 years of age) after cardiac surgery in infancy. Subjects who were ≤6 months of age and undergoing major surgical treatment of CHDs with cardiopulmonary bypass with or without deep hypothermic circulatory arrest (DHCA) were eligible for enrollment. Exclusion criteria for the original cohort included: (1) multiple congenital anomalies, (2) recognizable genetic or phenotypic syndrome, and (3) language other than English spoken in the home.

Of the 550 subjects initially enrolled in the study, 381 returned for 4-year neurodevelopmental follow-up. Of those not returning, 64 subjects were deceased and 105 individuals were lost to follow-up. Of these 381 eligible subjects, an additional 51 individuals were excluded due to the possible presence of a genetic or chromosomal syndrome as evaluated by a senior board-certified medical geneticist. As well, 14 subjects lacked *APOE* genotypes, other covariate data, or neurodevelopmental outcome phenotype data and were therefore removed from consideration in the analyses, leaving a total of 316 subjects for genome-wide analysis.

### Genetic Evaluation

Subjects were evaluated by a genetic dysmorphologist at the 1-year and/or 4-year evaluations, with additional clinical genetic evaluation or testing performed as indicated. Due to the difficulty in recognizing neonatal dysmorphic features, some subjects were enrolled for whom a diagnosis of a genetic syndrome was made at a later date. Subjects were classified as either: having no definite genetic syndrome or chromosomal abnormality (normal), suspected genetic syndrome (suspect), or a definite genetic syndrome or chromosomal abnormality (genetic). Following this classification, each patient’s genetics records were individually reviewed by a second senior board-certified medical geneticist, also blinded to the GWAS data, to determine whether subjects were to be included or excluded from the current analysis, which focuses on nonsyndromic subjects. Due to this review, 51 subjects with known or suspected genetic abnormalities were excluded from analysis due to the potential for genetic confounding effects.

### Operative Management

Details about the operative management of patients in this cohort have been previously reported [7], [10], [22], [23].

### Data Collection

Data on preoperative factors that might affect neurobehavioral outcomes independently, including gestational age, birth head circumference, and birth weight, were obtained from hospital records. Weight and age at surgery were recorded for the initial operation and for subsequent procedures with cardiopulmonary bypass. Operative variables were recorded, including the durations of cardiopulmonary bypass and DHCA, lowest nasopharyngeal temperature, and hematocrit level after hemodilution [7], [10], [22], [23]. Demographic and clinical characteristics of the cohort are presented in **Table 1**.

**Table 1:** Baseline and operative characteristics of the cohort.

Baseline Characteristics	Cohort Subset (n = 316)
Gender, n (%)	
Female	136 (43.0%)
Male	180 (57.0%)
Ethnicity, n (%)	
Asian/Pacific Islander, Hispanic, or other ancestry	37 (11.7%)
African ancestry, not Hispanic	70 (22.2%)
European ancestry, not Hispanic	209 (66.1%)
Gestational Age, mean ± SD, weeks	38.6±1.93
Birth weight, mean ± SD, kg	3.18±0.59
Birth head circumference, mean ± SD, cm	33.7±1.98
*APOE* Genotype, n (%) a	
ε2	43 (13.6%)
ε3	187 (59.2%)
ε4	86 (27.2%)
Diagnostic Class, n (%)	
I: 2 ventricles, no arch obstruction	160 (50.6%)
II: 2 ventricles, arch obstruction	36 (11.4%)
III: 1 ventricle, no arch obstruction	31 (9.8%)
IV: 1 ventricle, arch obstruction	89 (28.2%)
Specific CHD Diagnoses, n (%)	
Hypoplastic Left Heart Syndrome	87 (27.5%)
Tetralogy of Fallot	52 (16.5%)
Transposition of the Great Arteries	30 (9.5%)
Ventricle Septal Defect	28 (8.9%)
Ventricle Septal Defect, Coarctation of Aorta	15 (4.7%)
Single Ventricle	23 (7.3%)
Other Distinct CHD Diagnoses	81 (25.6%)
Preoperative intubation, n (%)	91 (28.8%)
Preoperative length of stay, mean ± SD, days	2.16±2.74
Age at first operation, mean ± SD, days	41.3±53.9
Weight at first operation, mean ± SD, kg	3.91±1.26
Total cardiopulmonary bypass time in first operation, mean ± SD, min	66.2±40.4
Use of DHCA, n (%)	180 (56.9%)
Total DHCA time in first operation, mean ± SD, min	39.8±16.7
Hematocrit level after hemodilution in first operation, mean ± SD, %	28.0±3.99
Postoperative length of stay after first operation, mean ± SD, days	10.70±9.91

Abbreviations: SD  =  standard deviation. DHCA  =  deep hypothermic circulatory arrest.

a *APOE* genotypes were categorized into three groups: ε2 (ε2ε2, ε2ε3, or ε2ε4), ε3 (ε3ε3), and ε4 (ε3ε4 or ε4ε4).

### Four-year Neurodevelopmental Examinations

Neurodevelopmental evaluations were performed between the fourth and fifth birthdays. Growth measurements (weight, length, and head circumference) were recorded. A health history was obtained, focusing on the incidence of interim illnesses, hospitalizations, neurologic events or interim evaluations, current medication use, and parental concerns about health. Parents were asked specifically whether they had ever been told that their child had autism, Asperger syndrome, pervasive developmental disorder not otherwise specified, or ADHD. Attention and other behavioral skills were assessed through parental report by using the Child Behavior Checklist for ages 1.5 to 5 years (CBCL/1.5-5) and the ADHD Rating Scale-IV, Preschool Version (ADHD-IV).

The CBCL/1.5-5 is a questionnaire used to obtain parental reports of behavior problems and prosocial adaptive skills demonstrated within the previous 6 months [8]. Responses are grouped to produce 7 narrow-band problem scores and 5 *Diagnostic and Statistical Manual of Mental Disorders, Fourth Edition* (DSM-IV) oriented scores. The pervasive developmental problems (PDPs) score is one of the DSM-IV oriented scores resulting from the CBCL/1.5-5 and has been previously utilized to identify preschoolers at risk for autism [24]. Other scores from the CBCL/1.5-5 include a total problem score and 2 broad-band indices (internalizing and externalizing problems). The CBCL/1.5-5 total problems score consists of the sum of the scores for the 99 specific problem items on the form plus the highest scores for any written-in responses to item 100. All raw scores were transformed to T-scores with mean = 50 and these T-scores were used as the outcomes of analyses. Of these CBCL/1.5-5 scores, five previously analyzed outcome scores (attention problems, internalizing problems, pervasive developmental problems (PDPs), withdrawn behavior, and total problems) [7] were tested for genome-wide association (see **Table 2**).

**Table 2:** Improved estimates for four-year neuropsychological outcomes in children with isolated congenital heart defects.

Domain	Test	Score, Mean ± SD	Not excluding all geneticsyndromes (n = 381)	Excluding all (likely)genetic syndromes(n = 316)
Social Skills	CBCL/1.5-5 PDPs	54.6±7.21	15%	9.5%
	CBCL/1.5-5 withdrawn	54.5±6.76	11%	8.5%
Attention	ADHD Rating Scale-IV Inattention	5.83±5.00	30%	24.7%
	CBCL/1.5-5 attention problems	53.8±6.10	12%	7.9%
	ADHD Rating Scale-IV Hyperactivity	6.84±5.20	22%	21.5%
Behavior	CBCL/1.5-5 total problems	47.7±11.15	18%	15.2%
	CBCL/1.5-5 internalizing problems	48.7±11.27	22%	19.3%

Abbreviations. SD  =  standard error. ADHD  =  attention deficit/hyperactivity disorder. CBCL  =  Child Behavior Checklist for ages 1.5 to 5 years. PDPs  =  pervasive developmental problems.

The ADHD Rating Scale-IV, Preschool Version, is an 18-item questionnaire utilizing a Likert scale (0– not at all, to 3– very often) that requires parents to rate the frequency of ADHD symptom occurrences, as defined by the DSM-IV [6]. This scale was specifically developed for children 3 to 6 years of age. Normative data was collected from a sample of 907 children. Mean scores (not transformed) are provided for inattention and hyperactivity/impulsivity. Both the raw ADHD-IV scores for inattention and hyperactivity/impulsivity were analyzed for genome-wide association (see **Table 2**).

Subjects were classified as having normal, at-risk, or clinically significant scores for both the CBCL/1.5-5 and ADHD Rating Scale-IV scales in accordance with prior published guidelines and reports [6], [7], [8], [9].

### Genotyping

Whole blood or buccal swab samples were obtained before the operation and were stored at 4°C. 550,000 SNPs were genotyped utilizing the Illumina HumanHap 550 k BeadChip performed at the University of Pennsylvania Center for Applied Genomics. Quality control was performed at the University of Washington. Copy number variation (CNV) data was not included in these analyses. The SNP data was filtered out for patient call rate <97% and genotype call rate <99%, minor allele frequency (MAF) cutoff of <0.5% (for power considerations), and Hardy-Weinberg equilibrium (HWE) p<10^−6^. After these filters, 514,139 SNPs remained with a genotyping rate of 99.772% for 478 subjects, of which 316 are included in these analyses, as detailed above.

### APOE Genotyping

Genomic DNA was prepared and was used for determination of *APOE* genotypes using a previously published method [10], [25]. *APOE* genotypes were classified into three groups as follows: ε2 (ε2ε2, ε2ε3, or ε2ε4) ε3 (ε3ε3), or ε4 (ε4ε3 or ε4ε4), and the *APOE* ε2 genotype was included as a covariate in the linear regression model for all phenotypes based on considerable prior published evidence within this cohort that the *APOE* ε2 genotype is associated with markedly detrimental neurodevelopmental outcomes [7], [10], [22], [23], [26], [27]. Nine subjects had the ε2ε4 genotype and were included in analyses grouped in the ε2 group, rather than excluded, due to the overall small sample size, prior work in this cohort demonstrating significant ε2 effects on outcomes but modest or null ε4 effects [7], [10], [22], [23], [26], [27], and because *APOE* was not a study endpoint.

### Analysis

All analyses were performed in PLINK [28], with graphics produced by R (http://r-project.org). Genotypes were coded using an additive model. Due to the mixed genetic descent of the cohort (see **Table 1** for demographic information, including genetic ancestry), the first 3 principal component eigenvectors from principal components analysis (PCA) were utilized to adjust for potential population stratification. Of the 7 neurodevelopmental outcomes tested (see **Table 2**), only three (ADHD-IV Hyperactivity/Impulsivity, CBCL/1.5-5 PDPs, and CBCL/1.5-5 Total Problems) had a genomic inflation factor lambda <1.03 and are presented, as the high genomic inflation factor in other phenotypes could reflect false positive results. The outcome of all linear regression analyses in the GWAS were the continuous score values from the CBCL/1.5-5 and ADHD-IV scales.

Linear regression was performed on each outcome to obtain a residual phenotype value, adjusted for the following potentially confounding variables: the first 3 principal component eigenvectors for race, gender, gestational age, birth weight, birth head circumference, *APOE* ε2 genotype, diagnostic class, preoperative intubation, preoperative length of stay, age at first operation, weight at first operation, total cardiopulmonary bypass time, use of DHCA, total DHCA time, hematocrit at first operation, and postoperative length of stay. These surgical covariates had previously been determined to influence outcomes [22], [23], [29]. The residual phenotype then underwent further linear regression for evaluation of genotype effects utilizing an additive model.

Maternal education and socioeconomic status are important modifiers of neurodevelopmental outcomes [30], [31]. However, they were not included as covariates in analyses due to a poor correlation with the specific outcomes of ADHD-IV Impulsivity, CBCL/1.5-5 PDPs, and CBCL/1.5-5 Total Problems.

## Results

Demographic and clinical characteristics of the study cohort of 316 subjects are shown in **Table 1**. **Table 2** displays the mean, standard deviation, and percentage of our 316 subjects who were either at risk or clinically significant for the selected five CBCL/1.5-5 and two ADHD-IV neurodevelopmental phenotypes that had been previously studied in this cohort [7]. In comparison to a prior publication from this cohort [7], this subset has a lower percentage of subjects at risk or clinically significant for all phenotypes, likely due to the exclusion of 51 subjects with likely or confirmed genetic syndrome.

**Figure 1:**
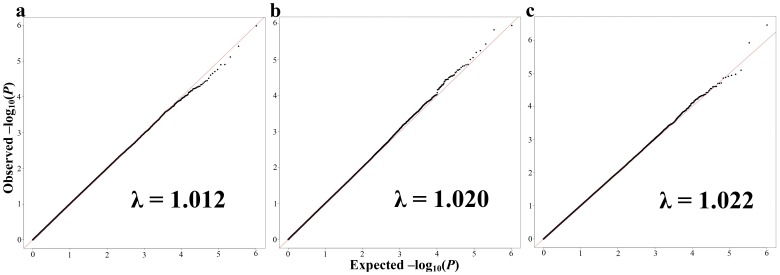
Genome-wide QQ plots of expected and observed –log(*P*) values for all SNPs analyzed in linear regression covariate-adjusted models for the selected four-year neuropsychological domains. Panel A presents the QQ plot for the phenotype, ADHD-IV Impulsivity Scale. Panel B presents the QQ plot for the phenotype, CBCL/1.5-5 PDPs. Panel C presents the QQ plot for the phenotype, CBCL/1.5-5 Total Problems. λ  =  genomic inflation factor.

**Figure 2:**
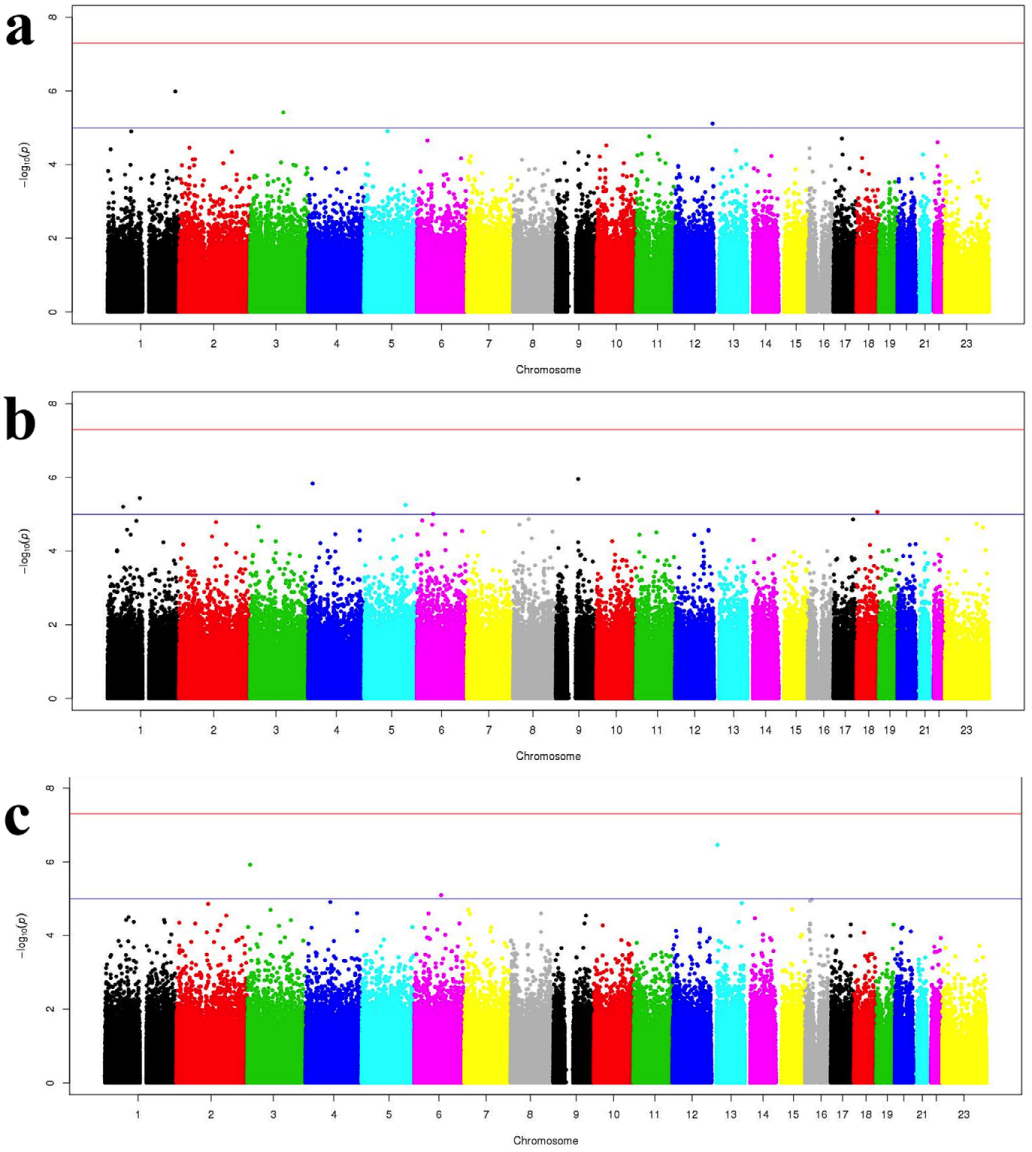
Manhattan plots of –log(*P*) for association of SNPs and chromosomal position for all SNPs analyzed in linear regression covariate-adjusted models. Panel A presents the Manhattan plot for the phenotype, ADHD-IV Impulsivity Scale. Panel B presents the Manhattan plot for the phenotype, CBCL/1.5-5 PDPs. Panel C presents the Manhattan plot for the phenotype, CBCL/1.5-5 Total Problems. Red horizontal line represents 5×10^−8^ threshold for genome-wide significance. Blue horizontal line indicates the 10^−6^ threshold for suggestive genome-wide significance.

Linear regression analyses of 514,139 SNPs in 316 subjects resulted in three adjusted phenotypes (ADHD-IV Hyperactivity/Impulsivity, CBCL/1.5-5 PDPs, and CBCL/1.5-5 Total Problems) passing our quality control for genomic inflation factor lambda <1.03 when adjusting for the first three principal components and the covariates described in the **Methods** section. **Figure 1A–C** displays the Q-Q plot of expected and observed log -10 p-values for the ADHD-IV Impulsivity (**1A**), CBCL/1.5-5 Scale for PDPs (**1B**), and CBCL/1.5-5 Total Problems (**1C**). For these three residual phenotypes, no SNPs had genome-wide significance of p<5×10^−8^. 10 suggestive SNPs had a p<10^−5^ and are presented in **Table 3**. Three SNPs with p<10^−5^ were found for ADHD-IV Impulsivity Scale, five SNPs for CBCL/1.5-5 PDPs, and two SNPs for CBCL/1.5-5 Total Problems Score. Manhattan plots of the –log *P* value for all 514,139 SNP**,** for ADHD-IV Impulsivity, CBCL/1.5-5 PDPs, and Total Problems, respectively, are presented in **Figure 2A–C**.

**Table 3:** Top SNP Results for Selected Four-Year Outcomes.

SNP	Chr	Position^a^	ClosestReferenceGene^b^	Distance to Closest Reference Gene	Minor/Major Alleles	MAF	EA only Betac,d	Beta^c^	EA only *P* d	*P*
**Top GWAS SNPs associated with ADHD-IV Hyperactivity/Impulsivity Scale** c										
rs4659682	1	233,008,289	*LGALS8*	–	G/A	0.042	2.146	2.313	2.33×10^−4^	1.03×10^−6^
rs7625411	3	114,294,118	*(GTPBP8)*	72,873 bp	C/T	0.177	2.956	2.779	4.98×10^−4^	3.84×10^−6^
rs543533	12	128,025,510	*NLRP9P*	–	C/T	0.377	−2.157	−2.07	9.67×10^−5^	7.67×10^−6^
**Top GWAS SNPs associated with CBCL/1.5-5 PDPs** c										
rs2261722	9	76,099,135	*PCSK5*	–	C/T	0.317	3.037	3.137	2.82×10^−5^	1.11×10^−6^
rs10516292	4	15,425,972	*(BST1)*	15,907 bp	C/T	0.219	3.688	3.92	2.63×10^−4^	1.47×10^−6^
rs11206315	1	54,455,429	*SSBP3*	–	G/A	0.157	4.264	3.982	1.01×10^−5^	6.25×10^−6^
rs12965975	18	71,438,271	*(C18orf62)*	169,694 bp	C/T	0.393	1.723	3.086	0.0277	8.68×10^−6^
rs228144	6	55,739,721	*BMP5*	–	C/T	0.112	3.142	4.407	3.23×10^−3^	9.83×10^−6^
**Top GWAS SNPs associated with CBCL/1.5-5 Total Problems** c										
rs11617488	13	21,091,962	*(FGF9)*	51,253 bp	C/T	0.275	−4.453	−5.245	5.13×10^−4^	3.47×10^−7^
rs9879307	3	9,845,065	*TTLL3*	–	C/T	0.317	−3.592	−0.2014	9.94×10^−3^	1.2×10^−6^

Chr  =  chromosome. EA  =  European Ancestry. GWAS  =  genome-wide association study. MAF  =  minor allele frequency. SNP  =  single nucleotide polymorphism.

a Position information and annotation from reference assembly 36.3.

b SNPs not found within a gene region are represented in parentheses, e.g. (*GTPBP8).*

c All beta-coefficients and p-values are the result of linear regression, where the outcome was the residual phenotype, adjusted for by the covariates listed in the methods.

d Analysis performed on the majority genetic descent group (EA) of the cohort (n = 209), adjusting for all covariates in the methods.

As this cohort utilized subjects of diverse genetic ancestry due to small sample size while adjusting for the first 3 principle components to correct for the inherent population stratification of this analysis, we sought to determine the effects of the 10 suggestive SNPs in the majority European ancestry (EA) subset (n = 209) of the cohort (see **Table 3**). All SNP p-values remained suggestive of significance. Sensitivity analyses between the EA subset and all other subjects of mixed genetic ancestry showed no significant differences in direction of SNP effect.

As previous studies into the genetics of ADHD have implicated *DRD4*, *DRD5*, and *DAT1* we explored their effects in our cohort. Rs3758653 and rs11246226 from our dataset were located near *DRD4* (upstream and downstream, respectively) and rs27072 was found within DAT1. No SNP tagged *DRD5*. None of these SNPs were associated with ADHD-IV Impulsivity in this cohort (p>0.10 for all three SNPs). While rs27072 is likely strong proxy marker for the VNTR associated with ADHD in *DAT1* as it is located 480 bases upstream of the VNTR, the cited VNTR in *DRD4* is located in the third (of 4) exon in *DRD4*, with neither of these two SNPs in strong LD with it. Thus, the 550 K data does not comprehensibly test the effects of *DRD4* or *DRD5* in this cohort.

## Discussion

Neurodevelopmental disorders are the most common sequelae following surgical palliation of CHD during infancy, with >30% of survivors scoring within the clinically significant range for attention problems in a recent study [32]. With improved surgical outcomes and techniques leading to vastly improved lifespan, a growing goal of independence has arisen in the families of nonsyndromic CHD subjects. However, while intellectual outcome measures vary from poor to excellent, CHD subjects have more problems with social involvement, school performance, and total competence in comparison to control subjects [33]. The Boston Circulatory Arrest Study reported that older post-surgical CHD subjects had difficulty integrating or coordinating the skills to accomplish higher-order goals, such as producing connected discourse or applying math concepts to solve problems [1], [2], [3], [29], [34], [35]. As a result, neurodevelopmental issues, with numerous effects across multiple spectrums of day-to-day life, can be a significantly limiting consequence in subjects who undergo surgical treatment of CHD.

We have completed the first GWAS for neurodevelopmental phenotypes in a unique cohort of 316 prospectively collected subjects, without known or likely genetic syndromes, who underwent cardiac surgery at less than six months of age. Although no SNP reached genome-wide significance (p<5×10^−8^); in total, 10 SNPs reached a threshold for suggestive significance (p<10^−5^) for one of three behavioral phenotypes: three for ADHD-IV Impulsivity, five for CBCL/1.5-5 PDPs, and two for CBCL/1.5-5 Total Problems.

This cohort is both the largest of its kind and also unique in its inclusion criteria, as it was designed to identify markers in genes that may affect the recovery of the brain after surgical palliation of a wide spectrum of CHDs and therefore adjusted for numerous potentially confounding inpatient and surgical variables, making it not possible to replicate these results in a similar, independent cohort. Of note, the Boston Circulatory Arrest Study had 155 eligible subjects and examined cognitive and behavioral outcomes after repair of transposition of the great arteries [1], [2], [3], [29], [34], [35]. As a result, we present our results as potential candidate genes involved in the pathogenesis of neurobehavioral problems in children who underwent cardiac surgery as infants, with the caveat that further investigation is required. However, interesting candidates did emerge.

The top SNPs for each outcome studied highlighted interesting candidate genes. Two SNPs, rs4659682 and rs2261722, associated with ADHD-IV Hyperactivity/Impulsivity and CBCL/1.5-5 PDP scores, respectively, were located in gene regions. Rs4659682 (p = 1.03×10^−6^ with ADHD-IV Hyperactivity/Impulsivity score) is a *LGALS8* intronic SNP, located in the 2^nd^ of 12 total introns. *LGALS8* encodes Galectin-8, a beta-galactoside-binding lectin with a conserved carbohydrate recognition domain [36]. The galectin family of proteins have been implicated in development [37], [38], growth regulation and apoptosis [37], [39], and other functions [36]. Rs2261722 (p = 1.11×10^−6^ with CBCL/1.5-5 PDP score) is a *PCSK5* intronic SNP, located in the 17^th^ of 35 total introns. *PCSK5* encodes a member of the subtilisin-like proprotein convertase family that processes proteins into their active forms, such as nerve growth factor [40]. As such, *PCSK5* is involved in neurodevelopment [40] and has been associated with a decrease in ventricular volume in a case-control cohort collected for Alzheimer’s disease and Mild Cognitive Impairment [41]. Finally, rs11617488 is an intergenic SNP (3.47×10^−7^ with CBCL/1.5-5 Total Problems), approximately 50 kb from *FGF9*. *FGF9* is a member of the fibroblast growth factor family of proteins and has broad range of activities, including CNS development [42], embryogenesis [43], cell repair [44], and cell growth [45]. Of the other SNPs that predicted outcomes at p<10^−5^, rs10516292, an intergenic SNP approximately 16 kb from *BST1* stands out for its potential relevance to neurodevelopment and neuroresiliency, due to numerous independent studies and meta-analyses associating *BST1* variants and Parkinson’s disease [46], [47], [48].

The three SNPs identified for association with ADHD-IV Impulsivity score have not previously been identified in prior GWAS for ADHD [19], [20], [49]. The lack of replication of our results in other published GWAS for ADHD likely reflects several differences between our study and the previously published ones. Foremost, the cohorts are fundamentally different, as our study was designed to identify genes that may affect neuroresiliency in children after surgery to repair CHDs, and therefore adjusted for numerous inpatient and surgical variables that could act as confounders. As an example, *APOE* effects on neurodevelopmental phenotypes previously identified in this cohort are not found in healthy children [7], [10], [50]. *APOE* effects on neurodevelopmental outcomes have been found in children with other stresses, including lead exposure, oxygen deprivation, malnutrition, and cerebral palsy [51], [52], [53], [54], [55]. Additionally, the analysis strategy is divergent, as Hinney et al., Neale et al., and Lasky-Su et al. all utilized case-control GWAS approaches, though Lasky-Su et al. did analyze quantitative measures of ADHD. Third, the phenotype definition varied, as Hinney et al. analyzed all 3 subtypes of ADHD (primarily inattentive, primarily impulsive/hyperactive, and combined types), while Neale et al. and Lasky-Su et al. utilized only combined type ADHD. Lasky-Su et al. utilized the Long Version of Conner’s Parent and Teacher Rating scales and results from Parental Accounts of Childhood Symptoms interviews as their quantitative outcomes. Fourth, the neurobehavioral phenotypic diversity is far greater in our cohort, with subjects that not only score in the clinically significant range for ADHD-IV Impulsivity score, but also for CBCL/1.5-5 PDPs and total problems, and other four-year neurodevelopmental phenotypes previously reported [7]. Thus, the independent results presented in this article could potentially indicate that unique genetic resiliency pathways impacting ADHD that are important when a similar environmental insult has occurred.

This study represents the first time within this cohort that we have re-estimated the prevalence of clinically significance for 4-year neurodevelopmental behavioral phenotypes without 51 subjects with possible or definite genetic syndromes. Excluding these children, we have noted a lower proportion of subjects that are clinically significant or at risk than reported previously [7]. Thus, the current data presented provides a better risk assessment for nonsyndromic CHD patients.

The CBCL has been the most commonly used instrument in studying neurodevelopmental outcomes in CHD subjects after surgery [8]. With regard to the phenotypes studied in this cohort, the CBCL/1.5-5 PDP score has been reported as having good predictive validity in screening preschoolers at risk for autism spectrum disorder [24]. In contrast, results using the CBCL/1.5-5 Total Problems score have not been widely reported. This is likely because it is a broad-band index representing a wide spectrum, thus having high sensitivity for neurodevelopmental problems but low specificity for any particular disorder.

This study has focused on exploring the genetic basis for the development of neurobehavioral disability in children following congenital heart surgery, while accounting for the known effects of *APOE*. The degree of disability is expected to be multifactorial, involving genetic and environmental factors, and particularly, their interaction. While we removed subjects with likely genetic syndromes, some infants may have had unrecognized genetic disorders that influenced both structural heart and neurodevelopmental phenotypes. Moreover, there is increasing evidence that brain development of some children with CHD is abnormal *in utero*, resulting in microcephaly [56] and a structurally immature brain at birth [57]. In addition to these genetic factors, the repair of CHD here included surgical intervention, which necessitated the use of cardiopulmonary bypass or deep hypothermic circulatory arrest. We have shown that many operative and pre- and post-operative factors predict variation in neurodevelopmental outcomes. [58], [59], [60]. The sum of these environmental factors is observed in neuroimaging and neuropathological studies that demonstrate ischemic periventricular white matter injury as a result of surgical intervention [61], [62]. By removing subjects with likely genetic syndromes and adjusting for the effects of *APOE* and surgical covariates, we focused our efforts not on identifying genetic variation that causes CHD, but on genetic variation that predicts neuroresiliency or lack thereof, in the face of enormous physiological stress. The genetic factors identified in these circumstances may not predict similar outcomes in cases where this physiological challenge was not present. This is the case for APOE genotype, which does not predict outcomes in children who are not exposed to such a challenge [50].

Some limitations of this study must be considered. First, the cohort is 66.1% of European ancestry. Although we utilized all subjects who met our criteria, including subjects of African and Asian ancestry while adjusting for the first 3 principal components, variants that do not occur in European ancestry subjects are underpowered here. As well, while the analysis strategy adjusted for population stratification within the cohort by adjusting for the first three principal component eigenvectors, there remains the possibility of false positive results. However, when we removed all genetic ancestries except for the majority European ancestry, we observed p-values that were suggestive of significance. Ideally, these GWAS results would be replicated in a separate and independent cohort to decrease false positive results. However, this was not possible in our unique cohort, and as a result we are presenting our results as candidate genes for the development of neurobehavioral phenotypes after neonatal cardiac surgery. It is possible that any true associations here, like the *APOE* association we previously reported, will be replicated in other cohorts with neurological insults [51], [52], [53]. Strengths of this unique cohort include the robust neurodevelopmental phenotyping, which allows for investigation of phenotypes not readily available from the electronic medical records or in other retrospective studies. As well, in comparison with the Boston Circulatory Study, which specifically focused on patients with transposition of the great arteries [1], [2], [3], [29], [34], [35], our cohort includes data across a spectrum of nonsyndromic CHD cases.

In conclusion, the suggestive results presented in this unique study offer candidate genes that may be implicated in the pathogenesis of neurobehavioral problems in a child as sequelae of neonatal cardiac surgery or other neurological insult. Given the potential long-term morbidity of these neurodevelopmental problems, further study of these candidate genes is warranted. Identification of genetic variation underlying differential susceptibility provides a window into novel pathways that may provide guidance in developing therapies and preventative strategies in children with CHD. As was the case for *APOE*, identified as a pediatric neuroresiliency gene in this cohort, these genes may affect outcomes of a broader range of neurological insults, such as lead exposure and hypoxia.
